# Mechanical significance of morphological variation in diprotodont incisors

**DOI:** 10.1098/rsos.181317

**Published:** 2019-03-27

**Authors:** Philip J. R. Morris, Philip G. Cox, Samuel N. Cobb

**Affiliations:** 1 Hull York Medical School, University of Hull, Hull HU6 7RX, UK; 2 Department of Archaeology and Hull York Medical School, University of York, York YO10 5DD, UK

**Keywords:** incisor, mechanics, morphology, diprotodont, rodents

## Abstract

All rodents possess a single pair of enlarged incisors that grow throughout life. This condition (diprotodonty) is characteristic of Rodentia, but is also found in other mammals such as lagomorphs, hyraxes, the aye-aye and common wombat. This study surveyed lower incisor morphology across extant diprotodonts to examine shape variation within and between rodents and other diprotodonts, and to determine if tooth shape varies in a manner predictable from mechanics. Six linear and area variables were recorded from microCT scans of the mandibles of 33 diprotodont mammals. The curvature of the rodent lower incisors, as measured by the proportion of a circle it occupies, was shown to vary between 20 and 45%, with non-Glires taxa falling outside this range. Relative lengths of the portions of the incisor within and external to the mandible were not significantly correlated when the overall size was taken into account. Cross-sectional geometry of the incisor was significantly correlated with the external length of the incisor. Overall, incisor morphology was shown to vary in a way predictable from ecology and mechanics, in order to resist bending. Among non-rodents, lagomorph incisors closely resemble those of rodents, and, relative to rodents, hyrax and wombat incisors are somewhat smaller but aye-aye incisors are much more extreme in morphology.

## 1. Introduction

Rodent incisors are some of the most unusual and highly specialized teeth seen in mammals. All rodents have an upper and lower pair of elongated and continually growing incisors. Each incisor grows throughout life in a curved (more specifically helical, based on observation of longer and overgrown incisors) shape, with odontogenesis taking place constantly at the base in order to balance the tooth material continually worn away at the tip through gnawing. The distribution of materials within rodent incisors is also unusual, with enamel being restricted to a layer along the labial surface of the tooth [1,2]. This enables a sharp blade to be maintained constantly at the incisor tip as the harder enamel wears away more slowly than the dentine beneath it [3]. The upper and lower incisors project a long distance posteriorly within both the cranium and mandible. Upper incisors reach as far back as the level of the first cheek tooth in most rodents, and even further back in some chisel-tooth digging mole-rats [4]. Similarly, lower incisors extend well beyond the mandibular premolars and molars, and even stretch into the condyle in some species.

Such highly specialized incisors, while being diagnostic of rodents, are not restricted to that order. The possession of enlarged (often continuously growing) incisors, here referred to as diprotodonty, is found in a number of other extant mammals, including the sister-group to rodents, Lagomorpha (hares, rabbits and pikas), and a range of more distantly related taxa including hyraxes (Hyracoidea), the aye-aye (*Daubentonia madagascariensis*) and the common wombat (*Vombatus ursinus*). The lagomorphs probably inherited their enlarged incisors from a shared common ancestor with the rodents [5], but the incisors of hyraxes, the aye-aye and the wombat, while similar in morphology to those of rodents, must have evolved independently in each order [3].

The long, curved incisors of diprotodonts are principally used in food acquisition, and also the processing of hard food objects, such as nuts, seeds and geophytes [6]. However, diprotodont incisors can also be used for a number of other mechanically demanding and specialized tasks, e.g. bark-stripping by aye-ayes [7], the felling of large trees by beavers [8] and the digging of burrows through hard soils by mole-rats [9]. Given the range of variation in tooth function in rodents, very little is known about the variation in the mechanically relevant shape (curvature, length, cross-section, etc.) and the corresponding mechanical performance of incisors in rodents and other diprotodont mammals. Chisel-tooth digging mole-rats are one of the few rodent groups in which incisor form and function has been well studied. There is a clear positive correlation between the radius of curvature of the incisors and cranial length across rodents in general, but species which dig with their teeth have much larger incisors relative to skull size [10]. In addition, several studies have noted that the angle at which the incisor emerges from the alveolus (incisor procumbency) is greater in chisel-tooth digging rodents [4,9,11–13].

In biomechanical analyses, biological structures such as long bone diaphyses and the mandibular corpora have been frequently modelled as beams owing to their similarity in shape and because of the relative simplicity that this approximation confers on the calculations [14–16]. Given its shape and the nature of the forces to which it is typically exposed, the diprotodont incisor can also be biomechanically approximated as a curved beam subjected to bending. Measures of cross-sectional geometry, particularly cross-sectional area (CSA) and second moment of area (SMA), are important in understanding the ability of a beam to resist bending [16,17]. CSA quantifies the amount of material found at a cross-section, whereas SMA indicates how that material is distributed relative to the loaded axis. The cross-sectional geometry of the rodent incisor has been shown to correlate with ecological traits that affect incisor loading such as diet [18] and habitat [10,19,20], and is a good predictor of maximum bite force [21].

The aim of this study is to determine whether the lower incisors of diprotodont mammals are similar in morphology across a wide range of taxa or if there is substantial shape variation within rodents and between rodents and other mammalian diprotodonts. This study will also assess whether the lower incisors of diprotodonts vary morphologically in a manner predictable from the mechanical loading they experience. Lower incisors were chosen as the focus of this study as they have been the subject of fewer morphological analyses than the upper incisors [4,10]. Three main hypotheses will be tested:

(1) *All lower incisors have the same two-dimensional shape in lateral view*. That is, assuming the curvature of the incisor to be constant along its length and therefore part of a circle (the helix is simplified as a circle for this study), it is expected that all incisors will form the same proportion of a circle (will subtend the same angle). This prediction is based on previous research showing that the upper incisors of rodents were very similar in shape across a wide range of species, all being approximately semicircular [10].
(2) *There is no correlation between the length of incisor within the mandible and the length of the part of the incisor not covered by mandibular bone*. This study assumes the external part of the incisor to act as a cantilever beam that is fixed at the level of the alveolar margin. Under this model, the length of incisor within the bone has no effect on the bending mechanics of the external part of the incisor, and thus, the two sections of the incisor will vary independently.
(3) *There is significant correlation between the length of the external part of the incisor and its cross-sectional shape, in particular CSA and SMA*. Both of these measures give an indication of how resistant to bending the incisor is, and so it is hypothesized that both metrics will correlate positively with external tooth length.

Each of these hypotheses will also allow differences and similarities in the form–function relationship of the lower incisors to be investigated between the rodent and non-rodent taxa.

## 2. Material and methods

### 2.1. Sample

The sample in this study comprised osteological specimens of the mandibles of 33 diprotodont mammals. These included 27 rodents, chosen to cover the majority of extant families, and six non-rodent diprotodont species: two lagomorphs (*Oryctolagus cuniculus* and *Lepus europaeus*), two hyraxes (*Dendrohyrax arboreus* and *Procavia capensis*), one primate (*D. madagascariensis*) and one marsupial (*V. ursinus*). All specimens except the capybara (*Hydrochoerus hydrochaeris*) were imaged using microCT scanning, resulting in isometric voxels with dimensions ranging between 0.02 and 0.14 mm. Owing to its large size, the capybara skull was imaged on a medical CT scanner with a resulting voxel size of 0.42 mm. A full list of specimens, the institutions from which they were borrowed, and the scanning parameters are given in electronic supplementary material, datafile S1.

**Figure 1.**
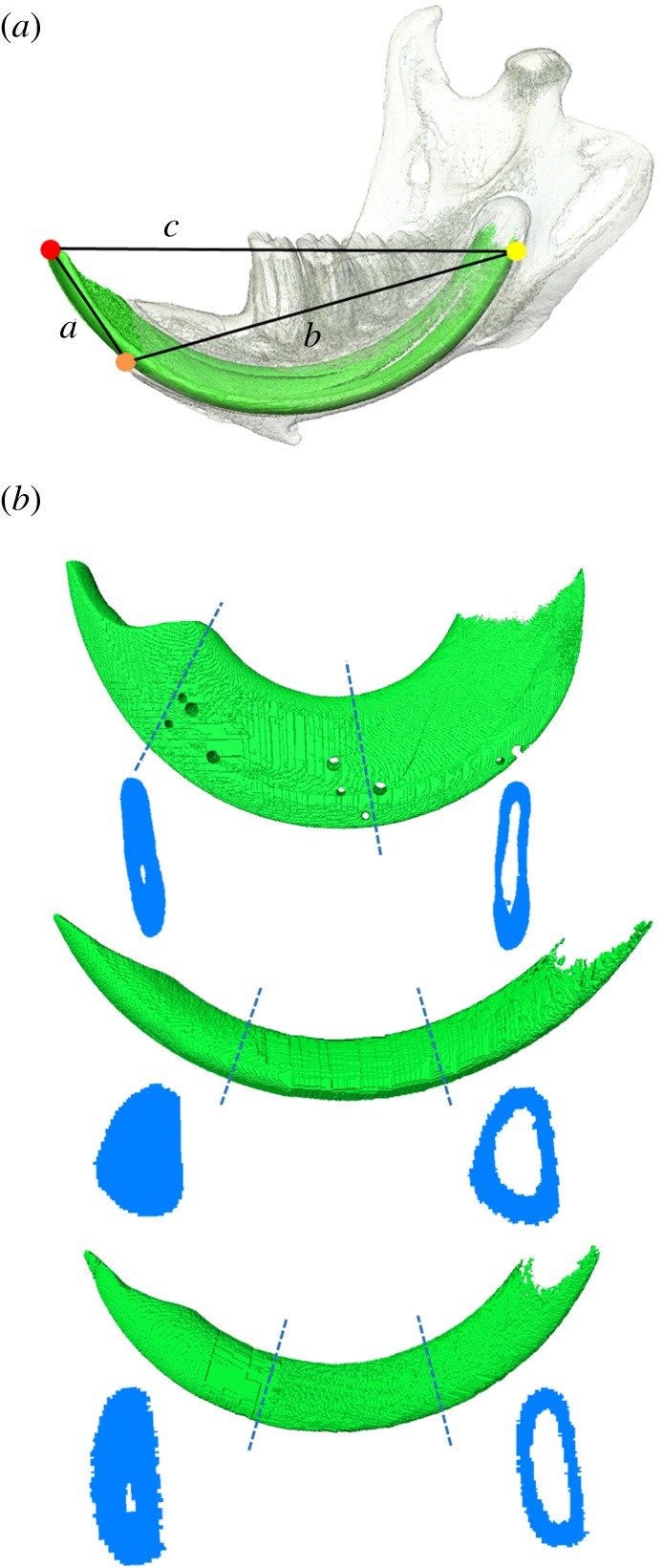
(*a*) Reconstruction of beaver lower mandible and incisor in lateral view showing landmarks and length measurements used to calculate incisor variables. Landmarks: red, incisor tip; orange, midpoint on labial incisor surface at alveolar margin; yellow, distalmost extremity of incisor. (*b*) Lateral view of reconstructions of the lower incisors of: *D. madagascariensis* (upper); *Rattus norvegicus* (middle); *Sciurus carolinensis* (lower), with cross-sections taken at the alveolar margin and at halfway along the internal incisor length (not to scale).

A hemi-mandible of each specimen was virtually reconstructed using Avizo 8.0 (FEI, Hillsboro, OR, USA), with the incisor being rendered as a separate object to the mandibular bone. A complete set of reconstructions is given in electronic supplementary material, table S1. Three landmarks were placed along the midline of the labial surface of the incisor (figure 1): one at the tip, one at the alveolar margin and one at the posterior extremity (here referred to as the base). These landmarks were used to align all incisor reconstructions to the same orientation and also enabled the calculation of six measurements from each incisor: (i) radius of curvature (*r*); (ii) total tooth length (TTL); (iii) internal tooth length (ITL); (iv) external tooth length (ETL); (v) CSA; and (vi) SMA. ‘Internal' and ‘external' tooth lengths here refer to the length of the portion of the incisor found within the dentary bone and the length of the portion protruding from the mandible, respectively.

*r,* TTL, ITL and ETL were derived by treating the three landmarks as the vertices of a triangle and calculating the lengths of its sides *a*, *b* and *c* (figure 1). *r* is the radius of the circle that fits the three landmarks and was calculated using a modified version of Heron's formula as in [4]

$$r=\frac{abc}{\sqrt{\left(2a^{2}b^{2}+2b^{2}c^{2}+2a^{2}c^{2}-a^{4}-b^{4}-c^{4}\right)}}.$$

TTL is the distance along the curve of the labial surface of the tooth between the tip and the base. It was determined by first calculating the angle subtended by the arc of the tooth (*θ*)

$$\theta =2\;\sin^{-1}\frac{c}{2r}.$$

This angle gave the proportion of the circumference occupied by the tooth, enabling its arc length to be calculated (assuming *θ* is in radians)

TTL= θr.

It should be noted that this formula is only correct for angles up to π radians, i.e. a tooth that encompasses less than half the circumference of a circle. As a check, the following value, derived from the cosine rule, was calculated (using the side lengths of the triangle in figure 1)

$$X=\;a^{2}+b^{2}-\;c^{2}.$$

A positive value of *X* indicated a tooth that encompassed more than half a semicircle, and thus, the calculated value of *θ* had to be corrected by subtracting it from 2*π*. The proportion of a circle occupied by the lower incisors was compared with that calculated for the upper incisors of a number of rodent species in a previous analysis [10]. Significant differences between the means and the coefficients of variation (CV) of the upper and lower incisors were tested using a *t*-test and a Fligner–Killeen test, respectively. Statistical analyses were carried out in PAST [22].

ETL and ITL (arc lengths from tip to alveolar margin, and from alveolar margin to base, respectively) were calculated by substituting *c* with *a* and *b* in the calculation of *θ*. The remaining two measurements, CSA and SMA, were determined from a cross-sectional slice taken through the incisor at the level of the alveolar margin. The slice was orthogonal to both the long axis of the incisor and the tangent plane at the alveolar margin landmark. The BoneJ module [23] of the ImageJ software [24] was used to calculate the CSA and SMA of the cross-sectional slice of the incisor.

**Figure 2.**
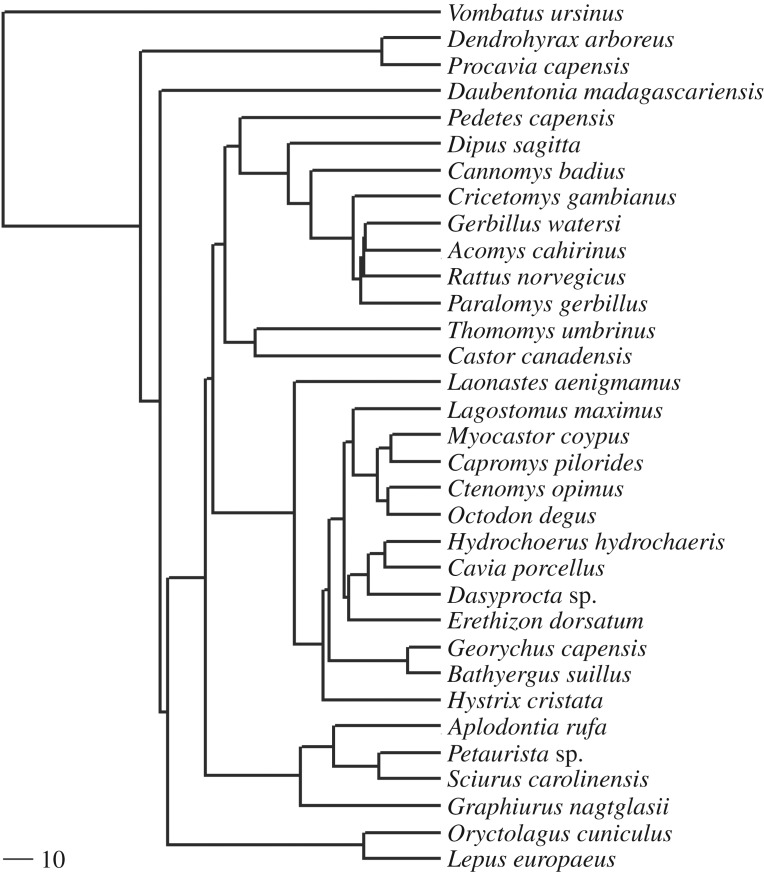
Phylogeny of species used in this analysis. Scale bar represents 10 million years.

The following bivariate plots were generated using the R statistical environment [25]: TTL versus *r*; ETL versus ITL; CSA versus ETL; and SMA versus ETL. In order to linearize the relationship between variables, the square root of CSA and the fourth root of SMA were plotted against ETL. To control for the confounding effects of size, ETL and ITL were also plotted against one another as fractions of circle. Phylogenetic generalized least-squares (PGLS) regression, implemented in the phytools package in R [26,27], was used to assess the relationship between the variables. A Brownian motion model of evolution was assumed and the underlying phylogeny, compiled using data from [28,29], is shown in figure 2.

## 3. Results

### 3.1. Incisor shape

**Figure 3.**
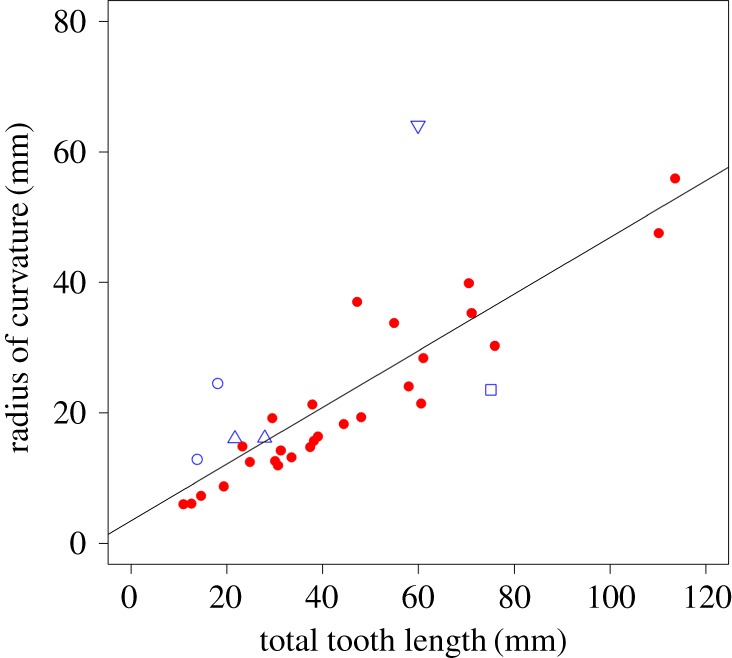
Scatterplot of radius of curvature against total incisor length. Red circles, rodents; blue symbols, non-rodent taxa; open square, aye-aye; open upward-pointing triangles, lagomorphs; open circles, hyraxes; open downward-pointing triangle, wombat.

The plot of *r* (radius of curvature) against TTL (figure 3) shows a clear positive correlation (*a* = 0.43, *R*^2^ = 0.65) between the two variables, which, after phylogenetic correction, is highly significant (*F* = 85.11, *p* < 0.001). However, although *r* generally increases as TTL increases, it can be seen in table 1 that there is a great deal of variation in the proportion of a circle encompassed by the incisor. Rodent lower incisors vary between 20 and 45% of a circle's circumference, with a mean of 34.2%, which is significantly different (*t* = 4.24, *p* < 0.001) from that of upper incisors (41.6%), as can be seen in table 2. Variability within the lower incisor sample (CV = 17.8) was greater than that of the upper incisor sample (CV = 14.2), but not significantly so, as demonstrated by a Fligner–Killeen test. Adding the non-rodents to the lower incisor sample extends the range further to 12 and 51%. Indeed, of the non-rodents, only the lagomorphs fall within the range of the rodents. The wombat and hyraxes have incisors that form a smaller proportion of a circle than rodents, whereas the aye-aye incisor forms a larger proportion. Hypothesis 1, that all lower incisors have the same two-dimensional shape in lateral view, is therefore rejected.

**Table 1.** Percentage of a circle encompassed by the lower incisors of rodents and non-rodent diprotodonts. Non-rodents in bold.

Species	%
***Procavia capensis***	**11**.**74**
***Vombatus ursinus***	**14**.**88**
***Dendrohyrax arboreus***	**17**.**08**
*Lagostomus maximus*	20.36
***Oryctolagus cuniculus***	**21**.**48**
*Cavia porcellus*	24.54
*Laonastes aenigmamus*	25.06
*Capromy spilorides*	25.94
***Lepus europaeus***	**27**.**49**
*Hydrochoerus hydrochaeris*	28.21
*Aplodontia rufa*	28.38
*Gerbillus watersi*	29.10
*Dipus sagitta*	31.83
*Acomys cahirinus*	31.99
*Myocastor coypus*	32.11
*Hystrix cristata*	32.38
*Paralomys gerbillus*	33.13
*Erethizon dorsatum*	34.27
*Rattus norvegicus*	35.06
*Graphiurus nagtglasii*	35.31
*Castor canadensis*	36.93
*Sciurus carolinensis*	38.05
*Georychus capensis*	38.17
*Pedetes capensis*	38.40
*Cricetomys gambianius*	38.73
*Thomomys umbrinus*	38.86
*Cannomys badius*	39.68
*Dasyprocta punctata*	40.00
*Petaurista petaurista*	40.42
*Ctenomys opimus*	40.64
*Octodon degus*	41.07
*Bathyergus suillus*	45.04
***Daubentonia madagascariensis***	**50**.**75**

**Table 2.** Summary statistics for percentage of a circle encompassed by the incisors of rodents. Upper incisor data derived from McIntosh & Cox [10]. ***p < 0.001.

	upper incisors	lower incisors	significance
mean	41.61	34.21	*t* = 4.24, ***
s.d.	5.91	6.08	*F* = 1.06, n.s.
CV	14.21	17.76	*T* = 13.71, n.s.

### 3.2. External and internal incisor length

**Figure 4.**
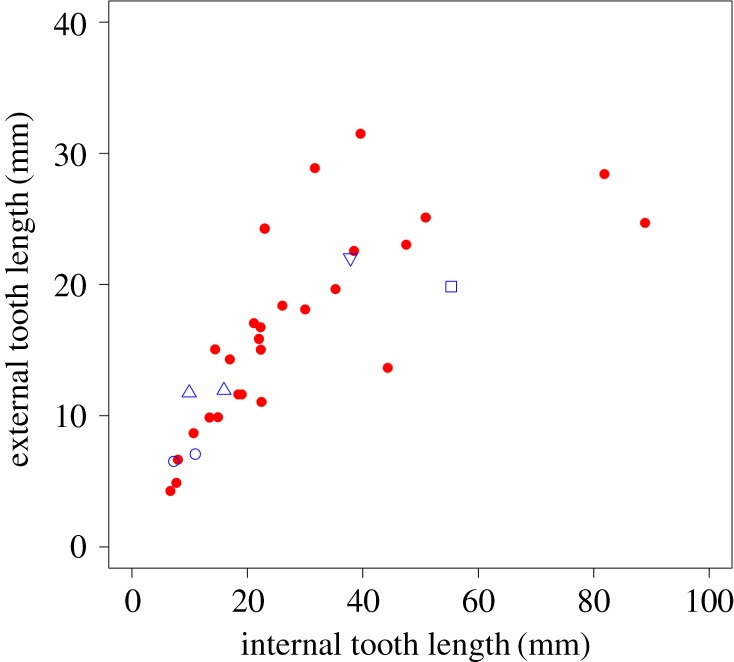
Scatterplot of external incisor length against internal incisor length. Red circles, rodents; blue symbols, non-rodent taxa; open square, aye-aye; open upward-pointing triangles, lagomorphs; open circles, hyraxes; open downward-pointing triangle, wombat.

**Figure 5.**
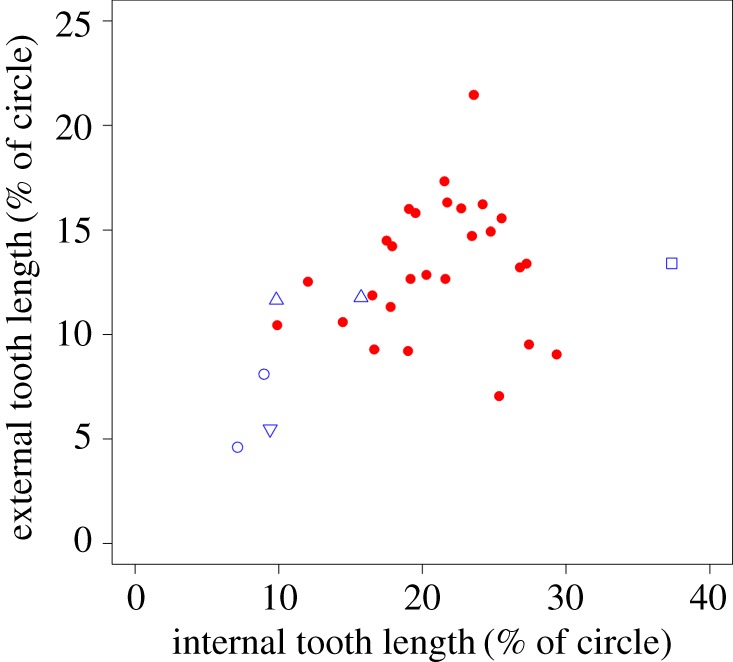
Scatterplot of external and internal incisor lengths expressed as fractions of a circle. Red circles, rodents; blue symbols, non-rodent taxa; open square, aye-aye; open upward-pointing triangles, lagomorphs; open circles, hyraxes; open downward-pointing triangle, wombat.

The plot of ETL against ITL (figure 4) indicates a relationship between these two measurements, but one that is potentially curvilinear rather than linear. At small sizes, ETL increases as ITL increases with a slope of 0.67. However, above an internal length of around 25 mm, the rate of increase in the external length slows dramatically, to a slope of 0.12, and scatter about the trend line increases substantially. Three rodent taxa (coypu, plains viscacha and Cape dune mole-rat) clearly plot above the curve and thus have longer incisors externally than would be predicted from the internal length of their tooth, whereas a rodent and a non-rodent taxa (springhare and aye-aye) are found below the curve, therefore displaying shorter incisors externally than expected. After phylogenetic correction, the log–log relationship between these two variables is highly significant (*F* = 60.27, *p* < 0.001). ITL and ETL were converted to fractions of a circle (by dividing by total circumference) and plotted against one another (figure 5). A PGLS model indicated that the relationship between these two variables was not significant (*F* = 2.14, *p* = 0.15). Hypothesis 2, that there is no correlation between the length of incisor within the mandible and the length of the part of the incisor not covered by mandibular bone, is therefore supported (with the caveat that there are some outliers among the rodent taxa).

### 3.3. Cross-sectional geometry

**Figure 6.**
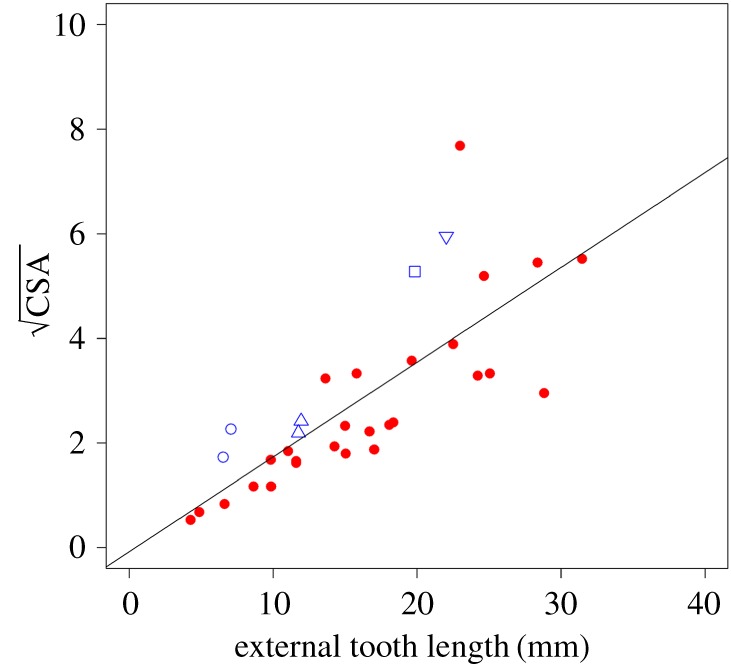
Scatterplot of square root of CSA against external incisor length. Red circles, rodents; blue symbols, non-rodent taxa; open square, aye-aye; open upward-pointing triangles, lagomorphs; open circles, hyraxes; open downward-pointing triangle, wombat.

**Figure 7.**
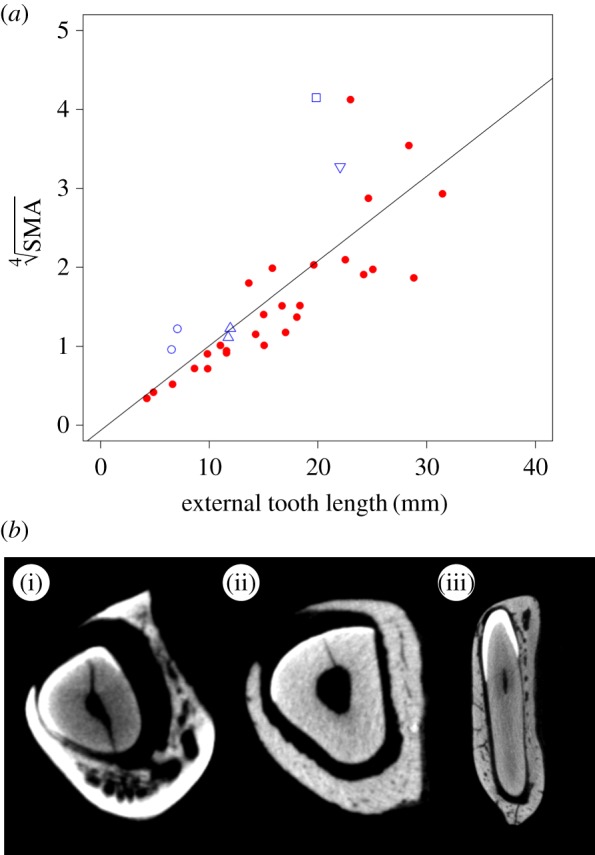
(*a*) Scatterplot of fourth root of SMA against external incisor length. Red circles, rodents; blue symbols, non-rodent taxa; open square, aye-aye; open upward-pointing triangles, lagomorphs; open circles, hyraxes; open downward-pointing triangle, wombat. (*b*) CT cross-sections of the incisor close to the alveolar margin illustrating the variation of cross-sectional geometry in the sample (not to scale). (i) *Hystrix cristata*; (ii) *Aplodontia rufa*; (iii) *D. madagascariensis*.

Both CSA and SMA show clear positive relationships with the ETL, as can be seen in figures 6 and 7 (CSA: *a* = 0.18, *R*^2^ = 0.61; SMA: *a* = 0.11, *R^2^* = 0.60). PGLS models indicate that these correlations are statistically significant (CSA: *F* = 0.50, *p* < 0.001; SMA: *F* = 0.55, *p* < 0.001). Three of the larger taxa (capybara, aye-aye and wombat) have a larger CSA and a larger SMA than would be predicted from the tooth length. On the other hand, the Cape dune mole-rat has a lower CSA and SMA than would be predicted from ETL. Hypothesis 3, that there is a significant correlation between the length of the external part of the incisor and its cross-sectional shape (as measured by CSA and SMA), is therefore supported.

## 4. Discussion

It can be seen from the results here that, on the whole, the lower incisors of diprotodont mammals vary in a predictable manner. There is a close correlation between the length of the incisor and its radius of curvature, between the length of the portion of the incisor within the mandible and the length of the exposed section, and between the cross-sectional morphology and the external length of the incisor.

### 4.1. Incisor shape

Previous research [10] found a close correlation between total curved length and radius of curvature of the upper incisor of a sample of rodents. Limited interspecific variation in the relationship between these two variables was previously noted, leading to the conclusion that most upper incisors approach a semicircle in shape [10]. The results here find greater variation in lower incisor shape (CV = 17.8) compared to upper incisors (CV = 14.2), albeit on a different sample of rodents, but indicate that this is not a significant difference. However, the proportion of a circle's circumference occupied by each incisor, given in table 1, does show a significant difference (*p* < 0.001) between upper (mean = 41.6%) and lower incisors (mean = 34.2%). This shows that, unlike the upper incisors, rodent lower incisors only approach a semicircle in a few taxa, and in most cases are substantially less than that.

It is not obvious why the shape of the upper incisor forms a greater proportion of the circumference of a circle than that of the lower incisor. One possible explanation is that the upper incisor is constrained to a particular shape because of a need to fit around the other contents of the rostrum—notably the nasal cavity and cribriform plate. Moreover, the upper incisor can, in most rodents, only stretch back as far as the beginning of the molar tooth row, but a relatively large amount of space is available in the dorsal axis, while the lower incisor can project backwards as far as, and in some cases into, the mandibular condyle, but has limited room to expand dorsally. Thus, by forming a semicircle, the upper incisor is maximizing its length in the space available and any increase in size will simply result in a larger semicircle, whereas the lower incisor forms a smaller part of a larger circle, and increases in size will tend to increase the proportion of the circle encompassed.

From examination of the distribution of species within table 1, the relative length of the lower incisor appears to be associated with diet and habitat. Those rodents with relatively short incisors (occupying less than 28% of a circle) tend to feed on fruits, leaves and grasses [6,30–32], which, while they may require substantial processing by the molar teeth, do not necessitate high incisor bite forces during their ingestion. On the other hand, those rodents with longer incisors, forming 36% of a circle or more, either regularly incorporate hard food items (e.g. roots, nuts, wood) into their diet [8,31,33–36] or live in a fossorial environment [9] which may lead to the ingestion of large amounts of grit. It therefore seems that rodents experiencing greater rates of wear tend to have incisors that form a greater proportion of a circle. This mirrors previous research showing that chisel-tooth digging rodents tend to have relatively larger upper incisors than non-tooth digging rodents [10]. Further work directly analysing the relationship between diet and mechanically relevant incisor morphology is required. While general, broad dietary categories (e.g. carnivore, insectivore, omnivore, generalist herbivore, specialist herbivore) are available in the literature for most of the taxa in this study, they do not provide information regarding the actual material properties (specifically the geometric and mechanical properties) of the foods and so are not relevant to understanding the mechanics of food acquisition and processing, and could generate misleading results. Unfortunately, the detailed information regarding the diets of these taxa, specifically the mechanical properties (e.g. Young's modulus of elasticity; hardness; toughness; fracture strength, etc.) and geometric properties (size and shape of the food items, and the implications for gape in the animal), necessary to carry out this analysis is not currently available and would require considerable effort to collect from the field.

### 4.2. External and internal incisor length

The second hypothesis of this study predicted that the length of the section of the lower incisor within the alveolus would not covary with the length of the portion external to the mandible. This prediction was based on the biomechanical assumption that the external part of the incisor acts like a cantilever beam fixed at the alveolar margin. As such, the length of the incisor within the mandible does not affect the ability of the external part of the incisor to resist bending. On first inspection, it seems that this hypothesis was not supported. There is a clear positive relationship between the two portions of the incisor (figure 4), although this relationship does not appear to be linear. As ITL increases above 25 mm, the rate of increase in ETL starts to taper off, and thus, the external part of the incisor is much shorter relative to the internal part in larger rodents. This interpretation should be treated with a degree of caution, though, as the trend may be driven by a small number of outliers and may reflect a weakening of the correlation between ITL and ETL as ITL increases.

It should be noted, however, that the relationship between ITL and ETL appears to be driven by overall changes in size. As the mandible gets larger, the entire incisor will also increase in size, and thus, the correlation between the lengths of the two parts of the incisor may simply reflect this. To account for the confounding factor of size, the ITL and ETL were converted to fractions of a circle by dividing them by total circumference. Under a PGLS model, it was found that the size-corrected ITL and ETL were not significantly correlated (figure 5), as predicted by the second hypothesis. It appears that the length of the external portion of the incisor can vary independently of the length of the internal section, and probably has done in response to the external forces experienced by the tooth. For instance, it can be seen that the taxa positioned below the curve in figure 4 tend to be those that engage their incisors in mechanically demanding activities such as gnawing roots and stems (*Pedetes* [36]), wood (*Castor* [8]; *Daubentonia* [7]) or bones (*Hystrix* [37]). These species probably have relatively shorter incisors externally, compared to other rodents, in order to resist the greater bending forces incurred during these activities. This also means that the perceived plateau of ETL noted above may be somewhat artefactual and driven by the unusually short external incisors of the beaver and porcupine.

It is also possible that the presence of the incisor within the mandibular body, in conjunction with the bony adaptations of the mandible, plays a role in the mechanical adaptation of the mandible to resisting bending during incisal biting, particularly in taxa which employ high force incisal biting. This hypothesis is the focus of a separate future study.

### 4.3. Cross-sectional geometry

As predicted by the third hypothesis, there is a significant positive correlation between both measures of cross-sectional morphology (CSA and SMA) and ETL. This fits with the biomechanical model of the lower incisor as a curved beam—as the beam gets longer, the bending moment will increase, and this can be resisted by increasing the amount of material in cross-section at the point of bending (the alveolar margin). In particular, the amount of material in the axis of loading (i.e. SMA) increases as the external length of the tooth increases. Such a relationship suggests that ETL can be estimated from cross-sectional geometry, which could be of particular use for the reconstruction of morphology in extinct rodents. The skulls and mandibles of fossil rodents often have broken or missing incisors (e.g. [38–40]) and it can be important to know their complete length for biomechanical analyses (e.g. [41]). The relationships shown here will enable such length estimations to be made. It should be recalled that the incisor is a composite structure (primarily dentine with a thin layer of enamel and cementum on the labial and lingual surfaces, respectively) which has been simplified for the purposes of this study as being composed of a single tissue. Additional work would therefore be required to determine if, in addition to facilitating the functional wear of the occlusal (biting) surface of the incisors, the enamel plays a mechanical role in stiffening the incisors.

### 4.4. Non-rodent diprotodonts

Six non-rodent diprotodont species were included in this analysis: two lagomorphs, two hyraxes, an aye-aye and a wombat, to determine if their lower incisors fall within the range of variation of rodent incisors for the metrics measured here. This is certainly the case for the lagomorphs, which fall within the range occupied by rodents for *r*, TL and the cross-sectional measures (figures 3–7). This is unsurprising, as lagomorphs and rodents are united within the clade Glires and are very likely to have inherited their enlarged incisors from a common ancestor [5]. However, it is not clear that the other non-rodents in this analysis are particularly similar to rodents with regard to their lower incisors.

It was found that hyrax incisors only partially resemble those of rodents. They show rodent-like proportions of the internal and external sections (figure 4), but plot a little way above the line with regard to their CSA and SMA relative to ETL (figures 6 and 7). In addition, among the hyrax taxa (figure 3), *Procavia* shows a larger *r* relative to TTL than other specimens in the analysis, although *Dendrohyrax* is similar to many rodents in this regard. Hyrax incisors are much shorter relative to overall mandible size, compared to the rodents (see reconstructed specimens in electronic supplementary material, table S1) and encompass a smaller proportion of a circle than any rodent in this analysis (less than 20%; table 1). This shortening results in relatively larger cross-sectional measures in both genera and a slightly enlarged radius of curvature in *Procavia*. Previous research [42] has indicated that hyrax incisors are used very differently to rodent incisors, functionally being more similar to canines, and this appears to be reflected in a somewhat different morphology.

Despite the large difference in body size, the wombat lower incisors are similar in a number of ways to those of the hyraxes. The arc of the incisor forms only 15% of the circumference of a circle—a value that is lower than any other rodent measured here and that sits between the two hyrax species. This results in the position of the wombat far above the line in the plot of *r* against TTL in figure 3. The proportion of ETL to ITL is similar to that of many rodents (figure 4), but its CSA and SMA are somewhat larger compared to ETL than most rodents (again like hyraxes). The relatively short incisors seen in the hyraxes and wombat are most likely a reflection of the diets of these species which are dominated by grasses and shrubs and do not include a high proportion of hard food objects [43–45].

The aye-aye is perhaps the most unusual species in this analysis. Its incisor forms just over a semicircle, which is a greater proportion of a circle than any rodent measured here (table 1). It also has a short ETL compared to ITL (figures 4 and 5), which, as mentioned above, is probably an adaptation to minimize bending stresses while gnawing into trees to gain access to wood-boring insect larvae [7]. The aye-aye has further strengthened its incisor by increasing the amount of tooth material in the axis of bending, so that, in cross-section, the aye-aye incisor is expanded labio-lingually, but reduced mesio-distally (figure 7*b*). This can be inferred from figures 5 and 6, which show that the CSA of the aye-aye incisor is relatively large compared to ETL (although no more so than that of the wombat), but that the SMA of the aye-aye incisor is enormous and sits the furthest above the line of all taxa, indicating the increase in size in the axis of bending. Overall, it appears that the highly unusual and specialized dietary ecology of the aye-aye has driven the evolution of an incisor morphology similar to but more extreme than that seen in rodents.

## 5. Conclusion

Overall, the lower incisors of rodents vary in a somewhat predictable way. The radius of curvature increases with the total curved length of the tooth, but there is some variation in two-dimensional shape, with rodent incisors varying between 20 and 45% of a circle. Relatively longer incisors are found in species that specialize in hard food items or have a subterranean lifestyle. The lengths of the portions of the incisor within and external to the mandible are also correlated, but this is largely an effect of overall size—when expressed as a fraction of a circle, there is no significant correlation between internal and external incisor length. As predicted by beam mechanics, the cross-sectional geometry is related to the external length of the incisor. Both cross-sectional measures (CSA and SMA) increase with increasing external length. Among non-rodents, only lagomorph incisors resemble those of rodents very closely. Hyrax and wombat lower incisors are somewhat foreshortened compared to rodents, whereas aye-aye incisors are elongated and specialized to resist the high bending forces generated by their bark-stripping behaviour.

## Supplementary Material

Incisor reconstructions

## Supplementary Material

Specimen information

## Data accessibility

Surface reconstructions or original microCT scans of all specimens are available from www.morphosource.org (specimen numbers given in electronic supplementary material, datafile S1).
