# Supplementary material for: Mechanical significance of morphological variation in diprotodont incisors
Source: R Soc Open Sci. 2019 Mar 27;6(3):181317. doi: 10.1098/rsos.181317 (PMC6458350; doi:10.1098/rsos.181317)
Supplement: Incisor reconstructions [file rsos181317supp1.pdf]

Species	Image of Mandible	Dietary Ecology
Acomys cahirinus **(Northeast African spiny mouse)**	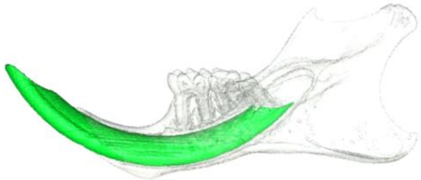	Omnivore – Seeds, fruits, insects, food scavenged from humans, shrubs (green leaves), molluscs, carrion.<br><br>Omnivore - (Nowak, 1999)
Aplodontia rufa (mountain beaver)	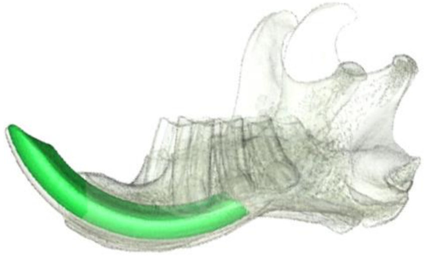	Herbivore – forbs, grasses, ferns.<br><br>Specialised Herbivore – (Samuels, 2009).
Bathyergus suillus **(Cape dune mole-rat)**	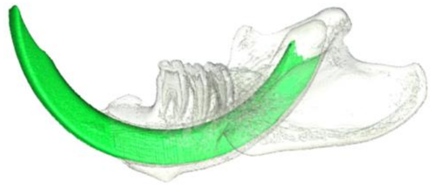	Herbivore – grass, sedge, roots, bulbs, tubers.<br><br>Specialised Herbivore – (Samuels, 2009).
Cannomys badius **(Lesser bamboo rat)**	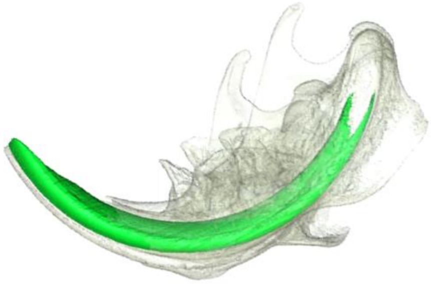	Herbivore – roots, bamboo, shoots, grasses. Occasional seeds and fruits.<br><br>Specialised Herbivore – (Samuels, 2009).
Capromys pilorides **(Desmarest's hutia)**	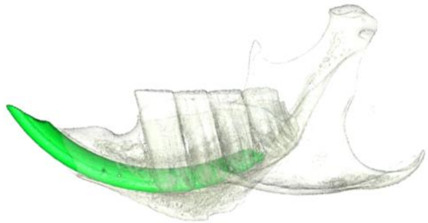	Omnivore – Bark leaves, fruits, small vertebrates, ground and tree level vegetation.<br><br>Omnivore - (Nowak, 1999).
Castor canadensis **(North American Beaver)**	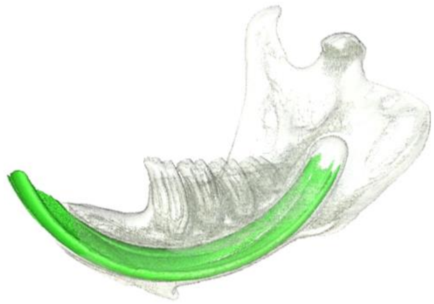	Herbivore – Leaves, bark, bud and roots, cambium (softer tissue of trees beneath bark).<br><br>Specialised Herbivore – (Samuels, 2009).

Cavia porcellus **(Domestic guinea pig)**	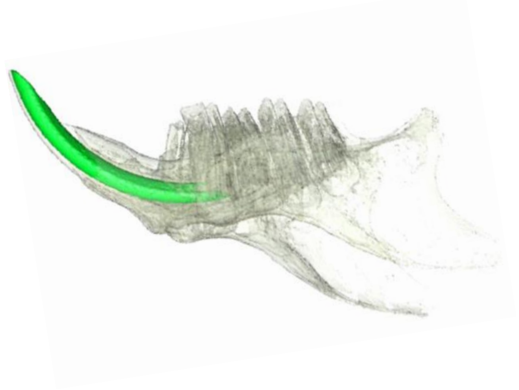	Herbivore – Leaves, roots and tubers, fruits, flowers, lettuce etc. (rely on humans).<br><br>Specialised Herbivore (*Cavia aperea*) - (Samuels, 2009).
Cricetomys gambianus **(Northern giant pouched rat)**	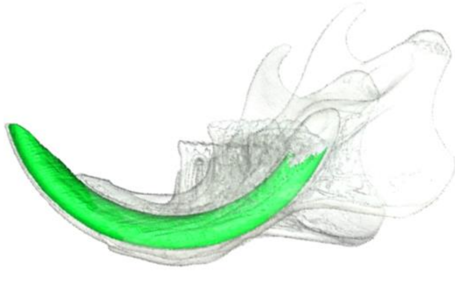	Omnivore – Fruits, vegetables, nuts, insects, molluscs, roots (sweet potatoes etc.).<br><br>Omnivore – (Nowak, 1999).
Ctenomys opimus **(Highland tuco-tuco)**	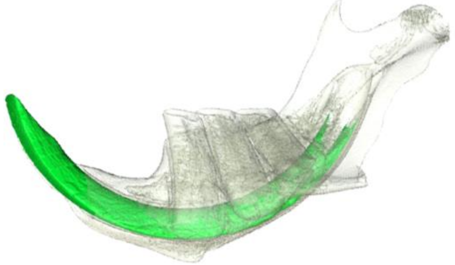	Diet for this species has not been extensively documented. Assuming that it is like other tuco-tuco, it is a herbivore – Grasses and roots primarily.<br><br>Specialised Herbivore (*Ctenomys conoveri*) - (Samuels, 2009).
Dasyprocta **(Agouti - species unknown)**	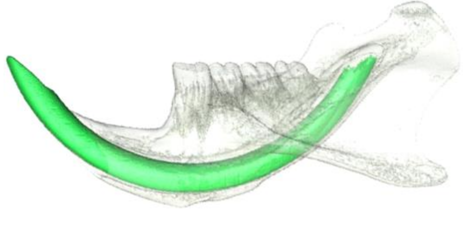	Species unknown. Assuming that it is like other Agouti it is primarily a herbivore – Leaves, roots and tubers, seeds, grains and nuts, fruits, occasional crustaceans.<br><br>Generalist herbivore – (Nowak, 1999) (fruits, vegetables, and various succulent plants)
Dipus saggitta **(Jerboa)**	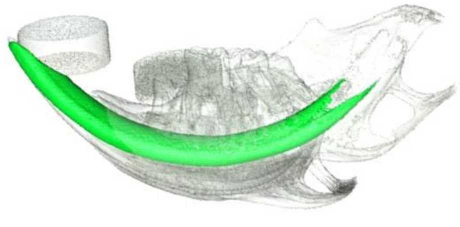	Assuming the specimen is *Dipus sagitta:* Herbivore – seeds, green plants. Some occasional use of insects.<br><br>Generalist herbivore (*Dipus [jaculus] aegypticus*) – (Samuels, 2009).

***Erethizon dorsatum* (North American porcupine)**	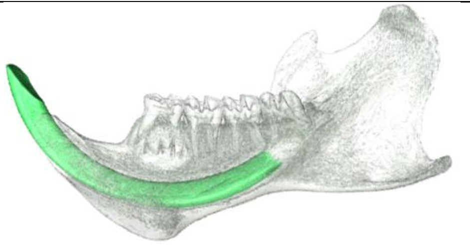	Herbivore – Bark, twigs, needles, buds, acorns, grasses, stems, flowering herbs, fruit.<br><br>Specialised herbivore – (Samuels, 2009).
***Georychus capensis* (Cape mole-rat)**	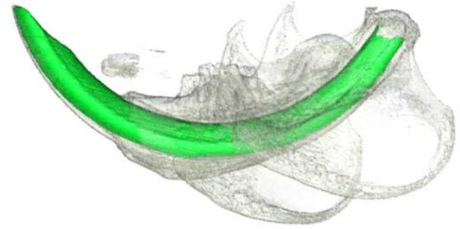	Herbivore – Green plant material and geophyte corms.<br><br>Specialised herbivore - (Samuels, 2009).
***Gerbillus watersi* (Waters gerbil)**	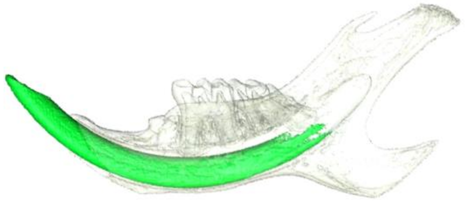	Diet not well documented – if like other gerbillines it tends to be herbivorous or omnivorous – eggs, insects, nuts, seeds, grasses, bulbs.<br><br>Omnivore (*Gerbillus paeba*) - (Samuels, 2009)
***Graphiurus nagtglassii* (Nagtglas's African dormouse)**	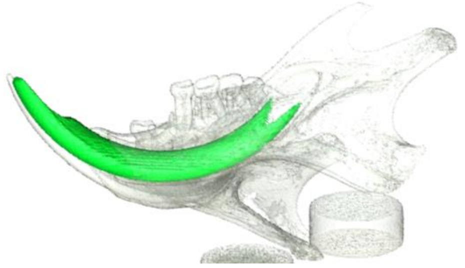	Diet for this species has not been extensively documented. Assuming that it is like other members of *Graphiurus* it is an omnivore – grains, fruits, eggs, insects, nuts, small vertebrates.<br><br>Omnivore - (Nowak, 1999).
***Hydrochoerus hydrochaeris* (Capybara)**	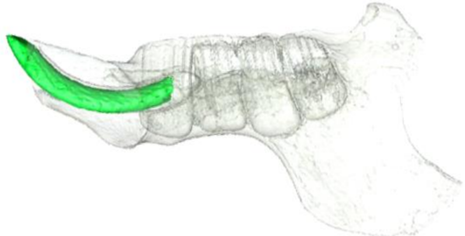	Herbivore – primarily grasses and aquatic plants. Occasionally eats bark and fruits. Coprophagy.<br><br>Specialised Herbivore - (Samuels, 2009).
***Hystrix cristata* (Crested porcupine)**	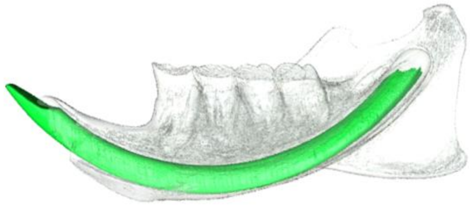	Herbivore – Bark, roots, tubers, rhizomes, bulbs, fruits, crops. Occasional insectivory and carnivory (small vertebrates and carrion – with some bone gnawing to sharpen incisors).<br><br>Generalist herbivore – (Nowak, 1999) (occasional insectivory).

***Lagostomus maximus* (Argentine plains viscacha)**	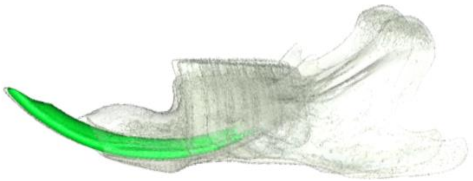	Herbivore – Seeds and grass. Will consume almost any vegetation when in captivity, however.<br><br>Generalist herbivore - (Nowak, 1999).
***Laonastes aenigmamus* (Laotian rock rat)**	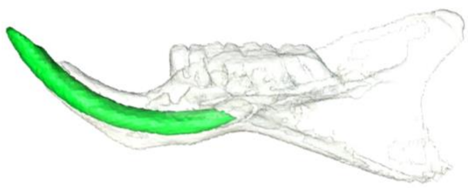	Herbivore – Leaves and fruits of tropical dicotyledonts (maybe also grasses according to its stomach shape).<br><br>Specialist herbivore – (Scopin, 2011) (primarily leaves).
***Myocastor coypus* (Coypu)**	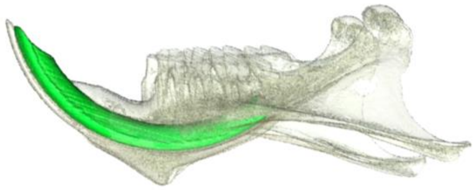	Herbivore – Primarily aquatic vegetation: stems, leaves, roots, bark.<br><br>Specialised Herbivore – (Samuels, 2009).
***Octodon degus* (Degu)**	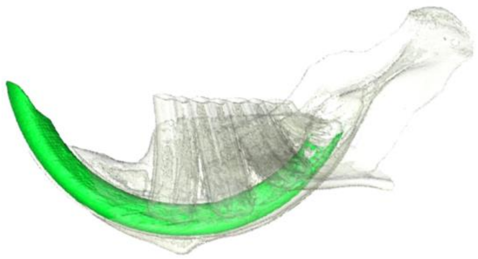	Herbivore – Grass, bark, leaves and seeds<br><br>Specialist herbivore – (Nowak, 1999).
***Paralomys gerbillus* (Gerbilline pericote)**	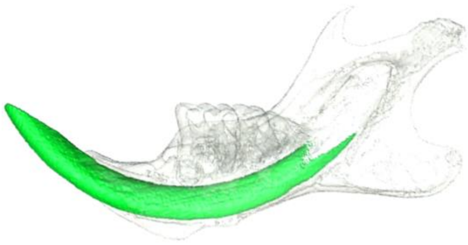	Diet for this species has not been extensively documented.
***Petaurista* (species unknown)**	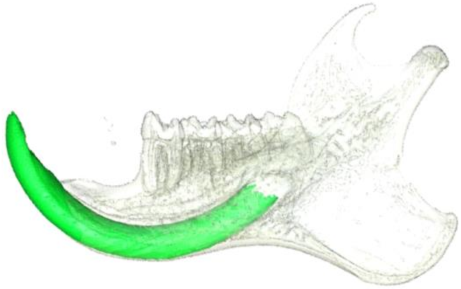	Generalist herbivore – (Nowak, 1999) (young leaves, tender shoots, fruits, nuts, flower buds).

***Pedetes capensis* (African springhare)**	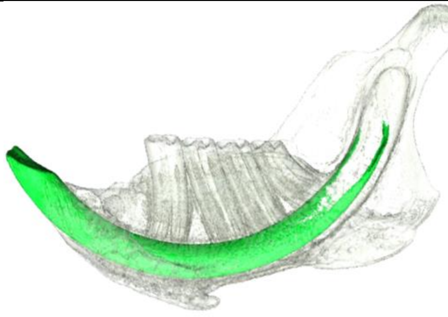	Herbivore/omnivore – barley, oats, wheat, with some occasional insectivory.<br><br>Generalist Herbivore – (Samuels, 2009).
***Rattus norvegicus* (Brown rat)**	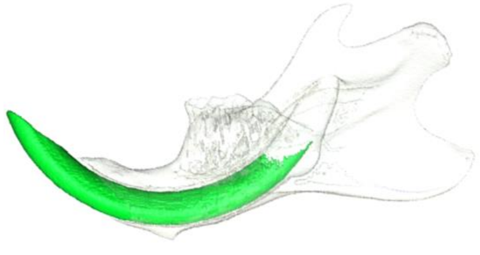	Omnivore – Birds, mammals and other vertebrates, insects and invertebrates, leaves, roots and tubers, fruit, grain, flowers, wood/bark, fungus, detritus.<br><br>Omnivore – (Samuels, 2009).
***Sciurus carolinensis* (Grey squirrel)**	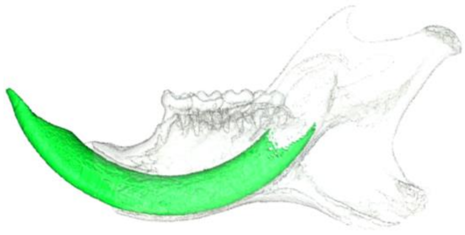	Omnivore – Birds, mammals, amphibians, eggs, carrion, insects, leaves, seeds, grains, nuts, fruit, fungus, bulbs and flowers, occasional cannibalism.<br><br>Omnivore (*Sciurus aberti*) - (Samuels, 2009)
***Thomomys umbrinus* (Southern pocket gopher)**	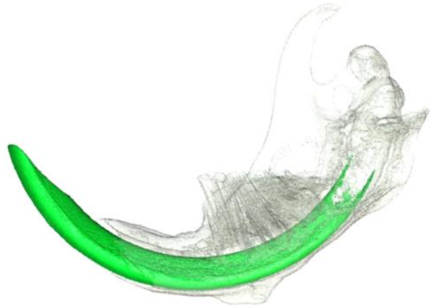	Diet for this species has not been extensively documented. If like other members of *Thomomys* they are generalist herbivores – leaves, roots, tubers, seeds, grains, fruit, grasses.<br><br>Specialised Herbivore (*Thomomys talipoides*) - (Samuels, 2009)

**Species**	**Image of Mandible**	**Dietary Ecology**
Oryctolagus cuniculus (European rabbit)	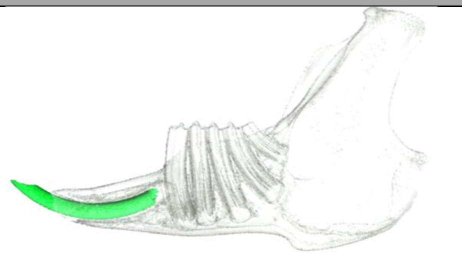	Herbivore – Grasses, leaves, buds, bark, roots. In captivity they are noted to eat lettuce cabbage, root vegetables, and grain.<br><br>Specialised herbivore – (Nowak, 1999; Matrai *et al.*, 1998).
Lepus europaeus (European hare)	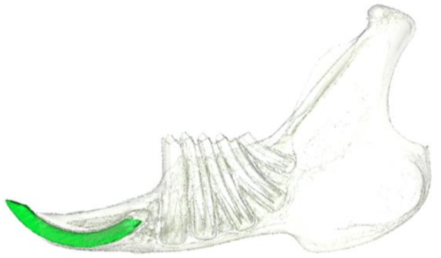	Herbivore – Grasses, herbs, field crops, twigs, buds, bark, coprophagia.<br><br>Specialised herbivore – (Nowak, 1999).
Dendrohyrax arboreus (Tree hyrax)	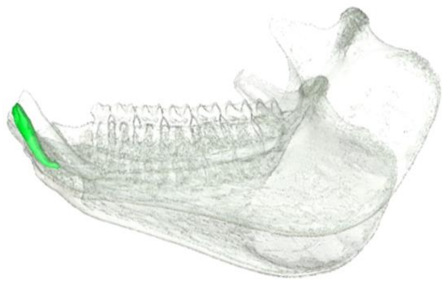	Herbivore – Leaves, twigs, shoots, fleshy fruit, hard seeds.<br><br>Specialised herbivore - (Nowak, 1999).
Procavia capensis (Rock hyrax)	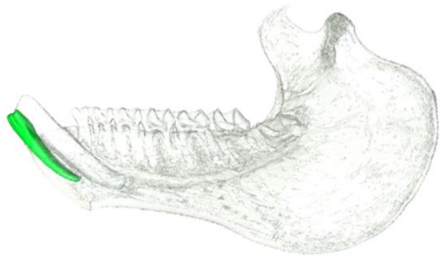	Herbivore – Lots of regional and seasonal vegetation, leaves, wood, bark and stems, fruit, berries, shoots, buds, leaves, bryophytes.<br><br>Specialised herbivore - (Nowak, 1999).
Daubentonia madagascariensis (Aye-aye)	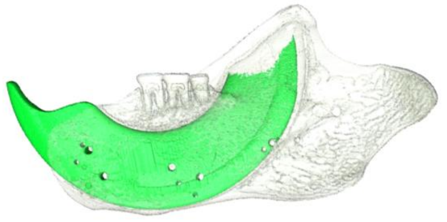	Omnivore – Seeds, nectar, fungus, and insect larvae. Access larvae through gnawing into woods to access xylophagous wood boring insects.<br><br>Omnivore - (Nowak, 1999).
Vombatus ursinus (Common Wombat)	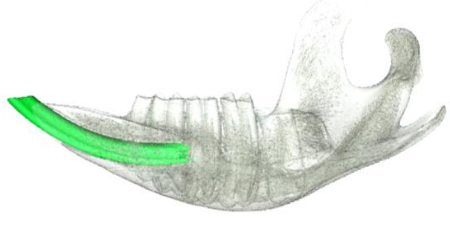	Herbivore – grass, roots and fungi. Prefers fresh seed stems (Nowak, 1999).<br><br>Specialised herbivore – (Nowak, 1999).
